# Experiences of registered nurses with regard to accessing health information at the point-of-care via mobile computing devices

**DOI:** 10.4102/curationis.v38i2.1498

**Published:** 2015-11-19

**Authors:** Esmeralda Ricks, Valencia Benjamin, Margaret Williams

**Affiliations:** 1 Department of Nursing Science, Nelson Mandela Metropolitan University, South Africa; 2 Campus Health Service, Nelson Mandela Metropolitan University, South Africa

## Abstract

**Background:**

The volume of health information necessary to provide competent health care today has become overwhelming. Mobile computing devices are fast becoming an essential clinical tool for accessing health information at the point-of-care of patients.

**Objectives:**

This study explored and described how registered nurses experienced accessing information at the point-of-care via mobile computing devices (MCDs).

**Method:**

A qualitative, exploratory, descriptive and contextual design was used. Ten in–depth interviews were conducted with purposively sampled registered nurses employed by a state hospital in the Nelson Mandela Bay Municipality (NMBM). Interviews were recorded, transcribed verbatim and analysed using Tesch's data analysis technique. Ethical principles were adhered to throughout the study. Guba's model of trustworthiness was used to confirm integrity of the study.

**Results:**

Four themes emerged which revealed that the registered nurses benefited from the training they received by enabling them to develop, and improve, their computer literacy levels. Emphasis was placed on the benefits that the accessed information had for educational purposes for patients and the public, for colleagues and students. Furthermore the ability to access information at the point-of-care was considered by registered nurses as valuable to improve patient care because of the wide range of accurate and readily accessible information available via the mobile computing device.

**Conclusion:**

The registered nurses in this study felt that being able to access information at the point-of-care increased their confidence and facilitated the provision of quality care because it assisted them in being accurate and sure of what they were doing.

## Introduction

The volume of health information necessary to provide competent health care today, has become overwhelming. Mobile computing devices (MCDs) are currently being introduced into health care settings to provide rapid access to the most recent clinical knowledge, making it an essential clinical tool for accessing health information at the point-of-care of patients (Stroud, Smith & Erkel 2008:31). Examples of MCDs include personal digital assistants, smart phones, tablets and lap tops.

The nursing field is a knowledge-intense area. Searching for nursing information can be a problem; accessing health information via the MCD makes it easier (Harkke 2006, cited in Axelson *et al*. 2007:611). The emergence of mobile computing technology has the potential to generate opportunities to enhance nursing care by providing access to clinical information at the point–of–care of patients (Axelson *et al*. 2007:611), thus allowing nurses to carry multiple references in their pockets, and to log onto clinical encounters at the push of a button (Leon *et al.* 2007, in Phillippi & Wyatt 2011:450). Whilst consulting patients, nurses are able to access drug reference and interaction applications, medical calculators, prescription rules, and specific topics in mobile computing devices (MCDs) such as editions of popular nurses’ reference manuals (Baumgart 2005:1217–1218).

The participants in this study all used MCDs (smart phones), with integrated cell phone functionality. In line with studies where nurses positively identified the MCD as being functional and useful in areas of drug reference, documentation, and access to patient data by offering portable and unobtrusive access to clinical data and relevant information at the point-of-care of patients, participants in this study concurred as to the efficacy of the MCDs (Lu *et al*. 2005:409). The advantage of using clinical data and relevant information at the point-of-care of patients is the improvement of care and the reduction of errors. Errors are minimised by preventing errors and adverse events with regard to the dosage of medication, facilitating a more rapid response of treatment after an adverse event has occurred, and by tracking and providing feedback on adverse events to other health care providers (Bates & Gawanda 2003:2526).

Various studies have been conducted in Africa and South Africa to demonstrate the usefulness of the MCD, where health care workers have enthusiastically embraced the use of the device and have successfully applied their newly gained knowledge in their health care practice. The main findings of these studies regarding the use of mobile technology as an information resource tool were that the MCD could positively change the way health care is delivered in the future (AED-SATELLIFE Centre for Health Information and Technology 2011:1).

## Problem statement

The researchers are registered nurses who were involved in an initial study in Nelson Mandela Bay Municipality (NMBM) regarding the use of MCDs for accessing information at the point-of-care to enhance clinical nursing practice. The initial study used a quantitative approach and highlighted that accessing information at the point-of-care via the mobile computing device enhanced clinical nursing practice in various ways. During the analysis of the open-ended questions of the above mentioned study, relating to the perceptions of the registered nurses whilst using the MCD for accessing information at the point-of-care of patients, the researchers identified a need to conduct a more in-depth study to provide evidence of effectiveness for the impact of MCDs on health knowledge, health outcomes and health care delivery (Blake 2008:165). Further recommendations from the previous study revealed research gaps relating to the extent to which the accessibility to information at the point-of-care enhances the effectiveness of patient care and nursing practice, and the impact of MCDs on the quality of care being rendered, measured as patient and staff satisfaction relating to the use of MCDs. The following research question guided this study: how do registered nurses experience accessing information at the point-of-care of patients via MCDs?

## Research objectives

This study explored and described the experiences of registered nurses with regard to accessing health information at the point-of-care of patients via MCDs within a hospital setting in the NMBM.

### Definition of key concepts

For the purpose of this study:

- A *registered nurse* is a person who is registered with the South African Nursing Council as a nurse, and who is qualified and competent to independently practice to the level prescribed (South Africa, *Nursing Act*, 33 of 2005).
- A *mobile-computing device*is a hand held computer that allows internet access (Stroud *et al*. 2008:31); in this study the device referred to is a smart phone that has been loaded with the Eastern Cape Disease Directory, the Standard Treatment Guidelines, an essential drug list, and a medical calculator.
- *Accessing health information* is a way of entering or reaching facts or details of the patients’ state of being free from illness or injury, or of the patients’ mental or physical condition, or facts that contribute and assist with the treatment of people who are ill or injured (Waite 2009:431).
- *Point-of-care* indicates the particular or precise moment when looking after the person who is receiving nursing treatment (Tulloch 1993:213).

### Contribution to the field

The emergence of mobile computing technology has the potential to generate opportunities to enhance nursing care by providing access to clinical information at the point-of-care of patients. The findings of this study highlight how searching for and accessing health information could be made easier through the use of MCDs.

## Research method and design

A qualitative, descriptive, explorative and contextual design was utilised (Fouche & De Vos *et al*. 2011:95-96).

### Population and sampling

The population comprised of 50 registered nurses employed at the public hospital who were trained in the use of the MCD in the initial study. The identified public hospital where this research was conducted is a tertiary-level health care facility in NMBM, Eastern Cape Province. The participants were interviewed within the physical environment and cultural context in which they worked.

Purposive sampling was used in selecting a research sample for the study. Purposive sampling is based on the researcher's judgement of the subjects that are typical, or representative of, the phenomenon being studied, wherein the researcher specifies the characteristics of a population of interest, and locates individuals who have those characteristics (Botma *et al*. 2010:126). Specific inclusion criteria included that they are registered nurses employed by the public hospital which participated in the initial research project, had been using the MCD for more than two years for accessing information at the point-of-care of patients and on a daily basis.

### Data collection

Permission to gain entry into the public hospital was obtained following approval from the Faculty Post Graduate Studies Committee (FPGSC) of the Nelson Mandela Metropolitan University (NMMU) and the Epidemiological Research Directorate, Department of Health, Bhisho. A pilot interview was conducted in which the information obtained was relevant to the study, no adjustments were required and the interview was used as part of the data collection for the study. Written consent was obtained prior to the interview. The interviews were recorded on a digital recorder, transcribed, analysed, and themes were formulated. These were then re-contextualised within the framework of the existing literature. Data saturation was achieved after ten in-depth unstructured interviews were conducted, beginning with the following question at each interview: ‘Tell me, how is it for you to access information at the point-of-care of patients via mobile computing devices?’

This question yielded rich, spontaneous descriptions of the phenomenon being investigated (Greeff 2011:343). Probing was used to obtain clarity (Kvale 2007:65). All interviews were scheduled during the participants’ on-duty time, as agreed to by the hospital management. The interviews lasted between 60 and 90 minutes. Comprehensive descriptive field notes were made after collecting information to make sense out of what had been observed (Holloway & Wheeler 2010:117). The data recorded were transcribed verbatim.

### Data analysis

Data were transcribed and analysed using Tesch's method (Creswell 2009:186). After coding the data, and developing themes, the researcher and the independent coder met in order for consensus to be reached with regard to the themes and sub-themes. Both the researcher and the independent coder agreed that data saturation had been reached, and that there was no need for further interviews to be conducted. The themes and sub-themes portrayed the storyline in a meaningful and descriptive way. Thick descriptions were used to convey findings and to bolster validity.

## Ethical considerations

Ethical clearance was obtained from NMMU (reference number: H11-HEA-NUR-002). The researchers ensured protection of the rights of the participants, that their participation was voluntary, that they were free to withdraw from the study at any time without penalty and that all information would be kept confidential. The participants were informed of the goal of the study, the voluntary and confidential nature of their participation, as well as of the possible benefits and outcome of the study following which each participant signed a written consent form. Confidentiality was maintained by removing from the research records any elements that could indicate the participants’ identities.

## Trustworthiness

To ensure valid results and trustworthiness the researchers used Guba's model as described by Krefting (1991:214). The focus of the model is truth value, applicability, consistency and neutrality. Credibility was assured through member checking and the use of peer examination, whilst confirmability was maintained through an audit trail.

Transferability refers to the ability to apply the findings of the study in another context or with other participants. The researchers used thick description, which was achieved by collecting and providing sufficient detailed data within the context of the study, purposive sampling and data saturation in order to ensure transferability.

The researcher responsible for the fieldwork ensured enhanced credibility through prolonged engagement with the participants, triangulation of data sources (interviewing the participants), and peer debriefing. Peer debriefing was carried out with a similar status colleague, outside the context of the study, who has a general understanding of the nature of the study and with whom the researcher could review perceptions, insights and analyses (Babbie & Mouton 2002). The measures to ensure dependability included code-recoded procedures, dense descriptions of research methods, triangulation and peer examination. Coding was carried out by an independent coder as well as by the researcher.

## Results

The participants comprised ten females from different race groups, aged from 39 to 53 years. Four main themes emerged from the data and each theme had a number of sub-themes (Table 1).

**TABLE 1:** Themes that emerged from analysis relating to the experiences of the registered nurses regarding accessing information at the point-of-care.

Themes	Sub-themes
Theme 1: Registered nurses expressed various experiences regarding the training received prior to implementing the MCD for accessing information at the point-of-care	Registered nurses experienced: 1.1 tension amongst the participants in the training sessions, 1.2 the training as enabling professional nurses to develop computer skills.
Theme 2: Registered nurses experienced a need for support in implementing the MCD for accessing information at the point-of-care	Registered nurses experienced a need: 2.1 to form their own support groups, 2.2 for continuous technical support.
Theme 3: Registered nurses experienced accessing of information at the point-of-care as beneficial for educational purposes	Registered nurses experienced accessing of information at the point-of-care as useful for: 3.1 educating patients and the public, 3.2 providing in-service education for registered nurses, 3.3 providing information to students.
Theme 4: Registered nurses experienced the access to information at the point-of-care as beneficial to patient care	Registered nurses experienced accessing information at the point-of-care as: 4.1 providing registered nurses with a wide range of accurate, readily accessible information, 4.2 allowing accurate nursing diagnoses to be made, 4.3 increasing the confidence of registered nurses in service delivery, 4.4 facilitating the provision of quality patient care.

MCD, mobile computing devices.

**Theme 1:** Registered nurses expressed various experiences regarding the training received prior to implementing the MCD for accessing information at the point-of-care. The various experiences expressed by the participants included feelings of tension because the training classes comprised individuals who had varying degrees of computer literacy, as well as feelings of enablement to develop computer skills.

**Sub–theme 1.1:** Tension amongst participants in the training sessions. The participants indicated that the registered nurses who had some previous computer training tended to grasp concepts faster than those who did not have any computer training or exposure to computers. Therefore the younger participants became irritated with the older participants because they were bored as a result of the slow progress with the training. The experiences of tension expressed by the participants are illustrated in the quotes below:

‘They could have moved faster, but now these people, they have to explain two, three times to them [*the older people*].’ (P1)

‘They should not put the old person, fifty years old with a twenty something year old. Obviously, there's gonna be much more advance with a twenty something year old.’ (P1)

The older participants felt left behind in the training, because they had not been exposed to computers before, thereby placing them at a disadvantage during the training with the younger participants, who are more advanced in computer literacy:

‘I found that the young sisters grasp information about computers easily. We were groups of the fifties and the thirties. The young ones were grasping information more easily than the old ones, so we were left behind.’ (P3)

**Sub–theme 1.2:** Training as enabling the registered nurses to develop computer skills. Some of the participants indicated that the two-day training has enabled them to develop computer skills because they were taught, for example, to conduct web searches for information, send and receive electronic mail, use the key pad to type, to download information from the web, and to perform calculations:

‘It [*the training*] enhanced qumy knowledge as far as computer literacy is concerned.’ (P8)

The registered nurses felt motivated to access the MCD because they learned much from the information that they accessed. Being able to use it for searching and acquiring information contributed to the registered nurses’ becoming comfortable with technology and expanding their use of technology to computers:

‘Having access to the mobile computing device can make you easier computer literate. You use it for all your things.’ (P1)

**Theme 2:** Registered nurses experienced a need for support in implementing the MCD for accessing information at the point-of-care. The need for support experienced by the participants included a need to form their own support group, and for continuous technological support from the information technology staff.

**Sub-theme 2.1:** A need to form their own support group. The registered nurses expressed their need to form their own support group which could be attributed to the fact that many of the participants were not computer literate and therefore encountered problems in using the MCD for accessing information at the point-of-care:

‘… [*W*]e assist each other if you are not able to get information.’

And:

‘… teach each other … you learn from each other.’ (P5)

Forming support groups with members who have more knowledge of computers, assists to explore the learning process:

‘… because they are computer literate in the group. So they were leading us all the time. So it was easy.’ (P6)

**Sub-theme 2.2:** A need for continuous technical support from the information technology staff. The registered nurses expressed their need for continuous technical support from the information technology staff during the implementation phase of the MCD for accessing information at the point-of-care. Their need for continuous technical support from the information technology staff could be attributed to the fact that they were not competent in using the MCD:

‘… [*H*]e [*the technical support staff*] had to do something about technology problems. They [*the technical support staff*] always help us a lot with the information and how to go about the phone.’ (P6)

**Theme 3:** Registered nurses experienced accessing information at the point-of-care as beneficial for educational purposes. The registered nurses in the study experienced accessing information via the MCD as important for educating the patients, the public, registered nurses and students.

**Sub-theme 3.1:** Accessing information via the mobile-computing device was useful for educating patients and the public. Participants indicated that accessing information via the MCD increased their knowledge, which enabled the registered nurses to answer questions and explain the management regarding the patients’ diagnosis:

‘The patient will be able to know more about their diagnosis, because the sister will be knowledgeable.’ (P7)

The aspect of the empowerment of the patient implies that the patient experiences a sense of authority because the registered nurses shared the information that they accessed via the MCD at the point-of-care with them:

‘You can’t do it [*educate*] if you don’t have the knowledge. We can help them [*the patient*] to the best of our ability to empower them.’ (P4)

The registered nurses indicated that the patients feel good because they are having their diagnosis discussed with them. The patients also feel good because the nurses value them by communicating to the patients with answers to their questions:

‘I could provide the patient with a quick answer regarding the treatment that he is on. It makes him confident about his treatment. So, really the phone is very informative, especially with us who don’t have the time to always go to the book for answers.’ (P1)

Participants expressed the need for accessing information via the MCD to educate the community, family and friends by providing them with information on rare conditions, their chronic conditions and acute conditions:

‘… [*E*]ven the community, if there is someone in the community that got a rare syndrome like the Marfan syndrome, then the family and friends ask you what it is; you don’t know at hand but you could be able to take it [*the MCD*] out of your pocket and look up [*access information*] on it, and always get more information to educate them [*the community*].’ (P2)

**Sub-theme 3.2:** Accessing information via the MCD was useful for providing in-service education for registered nurses. In-service education for registered nurses is important for the continuous growth and development of registered nurses. Nurses who do not update their knowledge on a regular basis could feel that they have become outdated, and have a need to learn new things:

‘Your job gets like a routine for you, you are a little bit outdated, you’ve just come on duty to do your daily task and until this device, what I experienced is, it brings me some sort of on-board [*with the latest information*].’ (P1)

Nursing is a dynamic profession, and nurses need to keep up to date with the changes that take place in nursing. Nurses have a constant need for new information, but it is not always possible for nurses to attend formal courses to keep themselves up to date with new developments, because of the constant shortage of staff:

‘It make me more knowledgeable, because you go for primary health care; a year course maybe, but the thing is we’re all human. You don’t know, not even a doctor knows all the specialties. I mean medicine is wide, and I mean that phone does not only cover urology, cardiothoracic, cardiology, ophthalmology; it covers everything.’ (P4)

The registered nurses in the study indicated that accessing information via the MCD assisted them in educating their colleagues with the latest information on diseases and their treatment through in-service training to their colleagues:

‘… [*A*]nd then they say, sister just check anything you can present to us [*registered nurses*], or any condition from your phone and then present it to us. So I’m always perusing and see and then I must present it now to the other nurses.’ (P9)

**Sub-theme 3.3:** Accessing information via the MCD was useful for providing information to students. The registered nurses experienced accessing information via the MCD as useful for providing information to students. There has been a need to educate the students, whilst they are practicing their clinical skills in the hospital:

‘We do have students. Like last week I was teaching them about type 2 diabetes. The way they were enthusiastic about the way I was giving them the information, because in the phone the information is straightforward. If you want the signs and symptoms, you will just go to the signs and symptoms.’ (P3)

‘Gone are the days when I had to tell them [*the students*] that I’m too busy to help them, or go to the books and look for the policy manual or procedure manual. I’ve got to go there and check and check and check there and page through it. I don’t even know which one, because it's a whole cabinet that is full of manuals. With this phone I save a lot of time, because I go straight to it and get the information.’ (P3)

The participants realised that it is their responsibility to teach the students of today who will be the registered nurses of the future. The quality of professional role models and teachings provided to the students to assist them in achieving professional maturity is of importance to their development and is part of the registered nurses’ accountability in the clinical setting.

**Theme 4:** Registered nurses experienced accessing information at the point-of-care as beneficial to patient care. The registered nurses in the study experienced accessing information via the MCD as being beneficial to patient care, by providing them with a wide range of accurate and readily accessible information at the point-of-care of the patient. The MCD allows accurate diagnosis to be made thereby reducing unnecessary referrals and costs. Other benefits experienced by the registered nurses include increasing the confidence of the registered nurses in service delivery, and the facilitation of the provision of quality patient care.

**Sub-theme 4.1:** Accessing information via the MCD provide registered nurses with a wide range of accurate and readily accessible information. The participants indicated that the wide range of accurate and readily accessible information was the latest information regarding the various conditions of the patients as well as the latest guidelines regarding the treatment of the conditions. The majority of the participants felt that it was easy to find the information on the internet:

‘It's got all the information you need. Your guidelines, it's there.’ (P1)

There has been a need for accessing information on human immunodeficiency virus (HIV) and tuberculosis (TB) because of the rapid infection rate and spread of these diseases. Many of the patients who are infected with HIV are co-infected with TB. The registered nurses experienced accessing information via the MCD as being able to provide them with information on the treatment of the aforementioned diseases, sometimes even before it has been published:

‘Before the minister announced it, the latest guidelines were on the phone … that the CD4 count should be 300 before starting ARVs.’ (P1)

‘Like I was excited when I received information on this phone that tells me that, did you know that how TB can be diagnosed within four hours, yet I was used to a long way of diagnosing TB on sputum and X-rays … and HIV as well. There's more information that I get updated with in HIV.’ (P5)

The availability of the latest information makes it possible to look at health practices elsewhere in the world. The registered nurses in the study could access information from outside South Africa through the help of Healthnet News via the MCD (AED-SATELLIFE 2011:1):

‘Before you knew it, it was there. It came through Healthnet News. It was there, nobody was aware of this H1N1 virus that is tackling us, and the treatment guidelines were there … the latest … You ask some information and then everybody who is connected up with this network responds to that information that you asked.’ (P1)

The convenience of accessing information via the MCD could be attributed to the fact that the registered nurses preferred to use it instead of books to access information:

‘The phone is better than the book, because you just go to where you want to go, and you get what you want to get immediately.’ (P8)

**Sub-theme 4.2:** Accessing information via the MCD allow accurate diagnosing to be made therefore reducing unnecessary referrals and costs. The registered nurses accessing information via the MCD experienced that it allowed accurate diagnosing by the primary health care nurses in the primary health care clinics, therefore reducing unnecessary referrals to the tertiary level hospitals. Primary health care clinics are often the first encounter that the patients have with health care. The registered nurses indicated that the patients in the community who are faced with health challenges have their first encounter with the primary health care nurses at the primary health care clinics. They also indicated that the government required that all health care be initiated at primary health care level:

‘You find that the person at home has got this challenge. The first person that he's going to see is the primary health nurse.’ (P3)

The staff capacity in the primary health clinics has been a challenge. Nurses are often left to provide the service with no access to doctors. The registered nurses indicated that accessing information via the MCD will assist nurses to make an accurate diagnosis although they do not have access to doctors:

‘… [*B*]ecause there are no doctors at times the nurses must do more primary health care. They’re seeing the patient without a doctor.’ (P1)

There has been a need for the primary health care nurses to make accurate diagnoses. Participants in the study indicated that access to information via the MCD allows for more accurate diagnoses to be made:

‘The nurses in the primary health care set up, they can access the phone and look at their guidelines to make a diagnosis … you can see the latest information and the latest guidelines.’ (P1)

The registered nurses in the study identified that the number of patient referrals to the tertiary level hospitals could be reduced if the primary health care nurses had access to information via the MCD. They indicated that the reduced number of patients referred to the tertiary level hospitals would allow for better quality care to be rendered to them:

‘A lot less patients will be sent to us [*tertiary level hospital*] because the information is there [*on the mobile computing device*]. There won’t be a need for them to send the patients to the hospital.’

And:

‘… because the [*referral*] numbers will definitely be lower. That means the quality care will be better.’ (P7)

The reduction in the number of patients referred to the tertiary level hospitals could contribute to saving costs for the department of health and for the government:

‘I think the better the knowledge and the better the treatment and not having patients to send to the hospital. It's transport costs that's being saved … so the whole of the Eastern Cape will benefit from the phone.’ (P4)

**Sub-theme 4.3:** Accessing information via the MCD increase the confidence of registered nurses in service delivery. Participants experienced accessing information via the MCDs as increasing their confidence in service delivery. The increase in confidence that the registered nurses experienced could be attributed to the fact that the accessed information made their work easier and, with the knowledge that they gained, they could confidently answer the patients’ questions:

‘This job with the mobile computing device, it makes the work lighter, really easier, and we are more confident in what we are doing … so it gives me that confidence.’ (P2)

Participants indicated that access to information contributed to the patients having trust in the registered nurses which could be attributed to the fact that the patients could see that the registered nurses were knowledgeable about their topics and that they were confident about what they knew:

‘… [*A*]nd then when you speak with the patients they can see on your face you know what you’re talking about, because you don’t tell them I have to phone the pharmacy.’ (P4)

Participants experienced accessing information via the MCD as contributing to their feelings of job satisfaction, which could be attributed to the fact that they felt happy to have access to information whilst they were on duty:

‘The knowledge makes me an effective nurse, because whatever I do, I do it for a reason and it gives me job satisfaction.’ (P3)

**Sub-theme 4.4:** Accessing information via the MCD facilitate the provision of quality patient care. The participants experienced accessing information via the MCD as facilitating the provision of quality patient care because they knew they were doing the right things for the patients, thus improving patient care:

‘So you check on this phone, then you find something and you apply it; it's good for the patient. I just feel that I’ve rendered a good quality care when the patient needed it. So it has improved the quality care of the patient.’ (P9)

Participants indicated that the provision of quality patient care could be attributed to the fact that the information on computers is regularly updated, meaning that the latest information is always available via the MCD:

‘We need upgrading to be always on the front side of things. Always having the latest things coming and the latest technology to be able to give quality care.’ (P1)

## Discussion

According to Voalte (2011:5), adequate provision for training and support needs to be made prior to using the MCD for accessing information at the point-of-care. This was reiterated by the participants who expressed relief at the fact that training had been provided to them prior to implementation of the project. However, it is clear that the training sessions that take place have to be carefully planned to avoid tensions arising amongst the participants as a result of the varying degrees of computer literacy. The participants indicated that the registered nurses who had some previous computer training tended to grasp concepts faster than those who did not have any computer training or exposure to computers. Therefore, the younger participants became irritated with the older participants because they were bored as a result of the slow progress with the training. This is echoed in a study by Nkosi, Asah and Pillay (2011:877) who observed that nurses with previous exposure to computers, and who have undergone computer training, are more confident, and have a positive attitude towards computers.

Participants indicated that the two-day training provided enabled them to develop computer skills. They were taught how to conduct web searches for information, send and receive electronic mails, use the key pad to type, to download information from the web, and to perform calculations. Brinkerhoff (2006:37) states that individuals become empowered when exposed to computer training. The Alexandria Proclamation indicates that computer skills empower people, making training a basic human right in a digital world (Sturgess & Gastinger 2010:195). The registered nurses in this study experienced their newly developed computer skills as enabling them to access information from the MCD with regard to various disease conditions.

According to Yaghmaie and Jayasuriya (2004:163), computer training and support groups are important factors when implementing the MCD for accessing information at the point-of-care, because training and support are necessary for the optimal and continuous use of the MCDs. Furthermore, support groups provide opportunities for users to participate in the process of information system development and successful performance, especially in areas such as health care facilities where the participants work.

Additionally, it is important to ensure technical support for those working with MCDs because using the device and fixing problems encountered is not comparable. As stated by Muchmore (2008:111): ’You are going to need technical support to fix the problems on your computer if you cannot fix it yourself.’.According to Bertolucci (2006:89) technical support is a guide to diagnosing some of the most common technical problems that befall a computer owner. Papafilippou, Templeton and Ali (2011:92) state that technical support from information technology staff is necessary as part of an approach to enhance the use of computers.

Registered nurses have a broad range of responsibilities, one of which is educating patients, the public, students and other registered nurses (Kudless & White 2007:36). Patient education is one of the aspects of patient care, and it forms an important part of total patient care. According to Visser, Deccache and Bensing (2001:4), the aim of patient education is to improve communication with the patient through a good nurse-patient relationship. Participants indicated that the patients felt good because the registered nurses explained their diagnosis and the treatment they would receive to them in detail.

South Africa has a quadruple burden of disease namely, HIV, non-communicable diseases (which include diabetes, hypertension and arthritis), communicable diseases (for example tuberculosis, malaria), and injuries because of accidents and violence. Providing the community with information regarding chronic and acute diseases could contribute to educating the population regarding their illnesses. This in turn could contribute to the reduction of the diseases profile of the country (Wei *et al*. 2011:29). Public education can improve health care status, reduce health care costs, and improve quality of life, and can be greatly enhanced by the use of mobile technology at the point-of-care of the patient.

Nursing is a dynamic profession which requires keeping up to date with constant changes. The constant addition of new information requires that nurses keep themselves up to date with new developments, but it is not always possible to attend formal courses. Participants experienced accessing information via the MCD, and therefore via the internet, plus the data added to the hard drive of these MCDs, as an opportunity to upgrade themselves with knowledge without having to leave their work places.

The confidence that the participants in this study experienced as a result of being able to access information at the point-of-care is echoed by Cockerham *et al*. (2011:231) who indicated that nurses are confident in their ability to deliver nursing care if they have access to the necessary resources. According to Lundberg (2008:86), confidence amongst nurses cannot be learned in the classroom; rather, confidence is acquired in the clinical setting by mastering newly learned skills, knowledge, and by experiencing success.

The work effectiveness of nurses is manifested in the quality of care received by patients (Purdy *et al*. 2010:903). Cullis and Webster (2010:269) suggest that best practice, which links to quality of care, means that the nurse stays up to date with the latest research, trials, guidelines and patient perspectives. Gadsby, Hall and Court (2010:1335) state that an online resource that offers a foraging service, surveying the literature and alerting clinicians to all the new important and useful information, enables the busy clinician to help keep up to date. Nursing, as a human phenomenon, is a specific type of interpersonal behaviour. Machines and computers contribute and assist the nurse in rendering patient care, thus enhancing the quality of the patient care (Mellish, Oosthuizen & Paton 2010:4). This was emphasised by nurses in this study who indicated that they had increased job satisfaction because of being able to offer quality care to patients as a result of their improved knowledge levels whilst accessing the MCD.

## Limitations of the study

Interviews were conducted at tertiary level facilities (hospitals) only. The study does not include the experiences of registered nurses in primary health care facilities.

## Recommendations

In light of the research findings and the indicated limitations, the following recommendations were formulated:

The management of public hospitals should be informed about the potential for the use of mobile-technology in health care settings so as to enable them to recognise the need for change as nursing care moves forward in the technological era. In-service computer education courses and workshops could be developed to enhance the computer skills of those who require assistance in using the MCDs. The researchers determined a need for further research relating to the effectiveness and impact of the implementation of the guidelines generated by this study.

## Conclusion

MCDs deliver ‘anywhere’ access to people and information. The volume of information needed to provide competent care in clinical practice has become overwhelming. The use of the MCD for accessing information could solve these clinical problems, hence the need to improve health care professionals’ access to this essential equipment. The health care industry is amongst the fastest growing handheld computing device markets, but in order for registered nurses to successfully utilise the MCD for accessing information at the point-of-care of patients, it is necessary to create an environment that is conducive to implementation. Furthermore, education is the most powerful means that can be used to bring about change. Enhancing the education of registered nurses, students and patients will give them a sense of authority because they will be well informed individuals regarding patient diagnosis and patient care. It is clear that the use of MCDs is essential to improve the quality of care for patients at the point-of-care, and more research is required to ensure that this occurs optimally, especially in the much needed primary health care settings.
